# Assessment of Knowledge, Attitudes and Practices Relating to Parasitic Diseases and Anthelmintic Resistance Among Livestock Farmers in Hamedan, Iran

**DOI:** 10.3389/fvets.2020.584323

**Published:** 2020-10-26

**Authors:** Alireza Sazmand, Golnaz Alipoor, Salman Zafari, Seyed Masoud Zolhavarieh, Abdullah D. Alanazi, Neil D. Sargison

**Affiliations:** ^1^Department of Pathobiology, Faculty of Veterinary Science, Bu-Ali Sina University, Hamedan, Iran; ^2^Department of Clinical Sciences, Faculty of Veterinary Science, Bu-Ali Sina University, Hamedan, Iran; ^3^Veterinary Council of Hamedan Province, Hamedan, Iran; ^4^Department of Biological Sciences, Faculty of Science and Humanities, Shaqra University, Ad-Dawadimi, Saudi Arabia; ^5^Easter Bush Veterinary Centre, Royal (Dick) School of Veterinary Studies and The Roslin Institute, University of Edinburgh, Midlothian, United Kingdom

**Keywords:** farmers' behavior, KAP questionnaire, parasite control, parasitic infection, one health

## Abstract

Iranian studies have shown a high prevalence of broad spectrum anthelmintic resistance (AR) in gastrointestinal helminths of ruminants. However, there is a lack of information about levels of knowledge, attitudes and practices among livestock farmers in Iran regarding the concept of parasite control and AR. This study aimed to evaluate the knowledge, attitudes and practices of livestock farmers of Hamedan, Iran, regarding parasitic diseases and AR by interviewing 150 farmers using a structured questionnaire. Most of farmers had some knowledge of the clinical signs associated with helminth parasitism, but more than half were unaware of the existence of zoonotic parasites. More than half of the participants had never heard about AR, but were interested to learn about it through their veterinarians. Those who were aware of the problem considered non-prescribed anthelmintic drugs to play a role in its emergence, while several of the participants believed that “more expensive” and “foreign-branded” drugs worked best. Almost all of the farmers reported that they frequently consulted with a veterinarian about anthelmintic treatments, but very few adhered to recognized principles of responsible and sustainable drug use. About half of the participating farmers treated their sheepdogs for helminth parasites, despite the common practice of regularly feeding likely infected livestock offal. Education had a significantly positive association with farmers' knowledge, attitudes, and best practice scores, while knowledge was significantly associated with both attitudes and practices. Based on these results, we recommend that regular country-wide classes should be held to educate farmers on the evidence-based principles of sustainable helminth control and prevention of zoonotic helminth diseases.

## Introduction

Helminth parasites are formost causes of endemic production-limiting diseases of ruminant livestock around the world (1). The development and availability of highly efficacious anthelmintic drugs has significantly contributed to reducing the economic burden of helminth parasitism. However, resistance has emerged to almost every anthelmintic drug group used in the control of nematode and trematode parasites infecting ruminants; and has become a serious economic problem for the livestock industry worldwide (2). The emergence of resistance in helminths of veterinary importance is largly influenced by host-related physiological and environmental factors that drive the parasites' life histories; but importantly the rate at which this occurs can depend on operational factors such as frequent prophylactic drug treatments, underdosing, and mass drug administration (3).

Progress in the development of socio-psychological research methods in behavioral sciences in recent years has created opportunities to study and inform animal health decision making (2). Information on knowledge (what is known), attitudes (what is thought), and practices (what is done) (KAP) is required to identify knowledge gaps and needs, and to understand factors and barriers that influence behaviors. This is a prerequisite for the planning and implementation of interventions (4). Questionnaire surveys of ruminant gastrointestinal nematode management practices have helped to inform our understanding the factors that influence farmers' behaviors in different specific regions (2, 5–8). This insight is necessary in order to optimize communication strategies and to improve compliance with specialist advice. However, the factors that drive farmers' decisions concerning sustainable helminth management will differ according to the society to which they belong (8).

Several Iranian studies have shown a high prevalence of resistance to broad spectrum anthelmintic drugs in gastrointestinal helminths of ruminants (9–12). However, information is lacking about levels of KAP regarding the concepts of parasite control and anthelmintic resistance (AR) among livestock farmers in Iran. This study was undertaken to assess the KAP of livestock owners with regards to parasite control and AR in the Hamedan province; with the aim of informing the development and implementation of sustainable control practices.

## Materials and Methods

### Data Collection

Hamedan province in the west of Iran has an area of almost 20,000 square km, with a population of about 1.8 million. It has a cold semi-arid climate with yearly rainfall of 384 mm and average temperature of 11.3°C. According to the latest official data in 2018, there were 371,000 cattle, 1,266,000 sheep and 108,000 goats kept in the province. As of May 2018, there were 303 registered livestock farms in the province (personal communication with Provincial Veterinary Organization). However, most animals are kept by landless smallholders and nomadic tribes. We hypothesized that most of our target population had not heard about AR and that their level of KAP would not be adequate in this context. Therefore, considering the awareness of farmers concerning AR as a main variable of interest, based on an expected 10% awareness in the population, desired absolute precision of 5% and a confidence level of 95%; the required sample size was calculated to be 138 (13). During June and July 2019, reputable veterinary clinics from different regions of the province were selected and study population was drawn from all farmers who referred to these clinics. Participants were provided with background information describing the team and study objectives. To reduce non- and biased- responses to sensitive questions, the participants were reassured that all responses would be anonymous. Hundred fifty farmers finally agreed to be interviewed.

### Questionnaire Design

A structured questionnaire was designed to collect data during interviews. The questionnaire was drafted (in Persian) and pre-tested by sending to five farmers and five veterinarians based in different regions of Hamedan province to evaluate its content and wording.

The final questionnaire contained four modules. In the first module, general questions, and socio-demographic information such as age, sex, education, years of experience in husbandry, herd size, and type of livestock were asked. Additional questions “have you already heard about AR?” and “if yes, from what sources” were included in this section to explore the general awareness of farmers about AR. To examine what farmers know about AR, how they think and how they behave in more depth, three other modules including 17, 6 and 10 items on knowledge, attitudes and practices, respectively, were designed (Supplementary File 1).

The knowledge section comprised of eight general questions about signs of parasitic diseases and nine more specific questions, such as: “does rotational use of anthelmintics prevent the emergence of AR?;” “is it necessary to consult with a veterinarian before deciding to use anthelmintic drugs?;” and “are parasitic worms zoonotic?.” Three possible answers (true/false or yes/no, and do not know) were provided for each question. Correct answers were scored 1 and wrong answers were scored −1. A zero score was considered for *I do not know* answers.

The questions in the attitudes section were designed to evaluate the way of thinking, or opinion of farmers concerning important issues in parasite control and AR mitigation, such as: the importance of non-prescribed anthelmintics in the emergence of AR; the seriousness of the problem of AR; and believes about effectiveness of more expensive and/or imported anthelmintics. Participants were asked to indicate their agreement/disagreement/no idea for each question. In this section, each response showing a positive attitudes toward the subject was given a score of 1, those answers indicating a negative attitudes were scored −1, and the *I have no idea* response was scored 0.

In the practices section, the frequency of farmers' self-reported practices regarding parasitic disease prevention and treatments was investigated. A five-point rating scale: *always*; *usually*; *sometimes*; *rarely;* and *never* was used. Ten questions were scored 4; 3; 2; 1; and 0, respectively, based on the concepts of best practice.

The farmers were asked a few additional questions at the end of the questionnaire: to identify routinely used anthelmintic drugs; to ask if they used medicinal plants for treatment of parasitic diseases; to ask if they had sheepdogs, and whether or not they have treated them with anthelminthic drugs; to determine how often they used anthelmintic drugs in their flocks or herds; and to ask what they would do if they became aware that the administered anthelmintics were ineffective. No score was assigned to these additional questions.

Two veterinary science students with backgrounds in livestock farming were chosen as interviewers, and were given 2 weeks of instruction prior to the interviews. A simple and brief concept of AR was explained to each farmer before the interview as “when antiparasitic drugs can no longer be effective on parasites, the term *anthelmintic resistance* is used.”

### Data Analyses

Survey data were entered by one of the investigators onto a Microsoft Excel (Microsoft Inc., Redmond, WA) spreadsheet and checked by another investigator. The data were then transferred to SPSS software (ver. 22.0, IBM, USA) for further analysis. Numerical, and categorical data were presented as mean and standard deviation (SD), and number and/or percentages, respectively.

The potential total score range for knowledge, attitudes and practices were between−10 and 10, −6, and 6, and 0 and 40, respectively. Spearman's rank correlation was used to determine the relationships of total knowledge, attitudes and practices scores with each other. In addition, Cronbach's alpha was calculated to measure the internal validity of questions in the knowledge, attitudes and practices section. Two cut-off points for knowledge and practices were determined using tertiles of the total score. Respondents were categorized into three nearly equal-size groups based on these cut-off points; namely poor, fair, and good for each section. For the attitudes section, participants were divided into two nearly equal-size groups based on the median of the total score; namely negative and positive attitudes groups.

The orderly categorized overall scores for knowledge and practices of farmers were used as the outcome variable. Univariable and then multivariable ordinal logistic regression analyses were used to evaluate the association of outcome variables with explanatory variables of age (four groups), years of experience in husbandry (two groups) and education level of the farmers (four groups), as well as the type of livestock (three groups) and herd size (three groups). Variables with a *P* < 0.2 in the univariable analyses were included in the multivariable models (14). The final models were constructed with a manual backward elimination procedure. Multicollinearity was evaluated amongst explanatory variables using Spearman rank correlation, and r_s_ ≥0.8 was considered as collinearity. A similar approach was used to determine the association of explanatory variables mentioned above with farmers' attitudes and their awareness of AR, using univariable and multivariable binary logistic regression analysis. In all analyses, a final two-tailed *P* < 0.05 was considered significant.

## Results

### Socio-Demographic Characteristics of Participants

Basic information relating to the respondents is shown in Table 1. One hundred and forty-eight (99.3%) of the enrolled participants were male, 36.7% were over 50 years of age and 62% had experience of over 10 years in domestic animal husbandry. More than half of the respondents (57.3%) had low levels of formal education (illiterate or primary school). Most (62%) kept sheep and goats, followed by cattle and a mixture of ruminant species.

**Table 1:** General characteristics of farmers (*n* = 150) in the study for assessment of knowledge, attitudes and practices regarding AR in Hamedan province, 2019.

**Demographic characteristic**	**Have heard of anthelmintic resistance**			
	**Number (%)**	**Yes**	**No**	***P*-value**
**Age (years)**				
<30	20 (13.3)	11	9	
31–40	35 (23.3)	17	18	
41–50	40 (26.7)	19	21	
>50	55 (36.7)	21	34	0.54
**Education**				
Illiterate	51 (34.0)	13	38	
Elementary	35 (23.3)	14	21	
Intermediate	36 (24.0)	22	14	
High school & higher	28 (20.0)	19	9	<0.001
**Years of husbandry-related work**				
Up to 10	57 (38.0)	25	32	
>10	93 (62.0)	43	50	0.777
**Type of livestock**				
Cattle	24 (16.0)	7	17	
Sheep and/or goats	93 (62.0)	44	49	
all of them	33 (22.0)	17	16	0.203
**Husbandry**				
Grazing plus stall feeding^a^	128(87.7)	61	67	
Stall feeding	18 (12.3)	4	14	0.042
**Herd size**				
≤10	27 (18.0)	4	23	
11–49	70 (46.7)	29	41	
≥50	53 (35.3)	35	18	<0.001

a *Pasture graze only mode was not practiced*.

### General Knowledge About AR and Source of Information

Less than half of the participants (45.3%) had heard of AR and were aware of it. Most of those who were aware had gained this knowledge from other farmers (55 respondents). One hundred and thirty six farmers (97.9%) expressed willingness to learn more about the concept of AR; most citing veterinarians as their main source of information on the topic. Other trusted sources of information were Ministry of Agriculture extension courses (85 respondents), TV/radio (33 respondents), internet and social media (23 respondents) and books or journals (20 respondents). Associations between awareness of AR and general characteristics of farmers are shown in Table 1. In the univariable analysis, education, type of livestock, husbandry and herd size had *P* < 0.2. In the multivariable binary logistic regression model with backward elimination, education and herd size remained in the final model (Table 2). The results show that the chance of awareness is increased 2 to 3 fold in farmers with higher education and larger herds.

**Table 2:** Results of logistic regression analysis for factors associated with awareness of AR in farmers, Hamedan province, 2019.

**Variables**	**Wald^a^**	**SE^b^**	**OR^c^ (95% Confidence Interval)**	***P*-value**
Constant	26.339	0.830	–	<0.001
Education	11.832	0.173	1.82 (1.29–2.55)	0.001
Herd size	16.143	0.297	3.29 (1.84–2.90)	<0.001

a Wald statistic;

b Standard error;

c Odds ratio.

*Education and herd size were treated as continuous variables*.

### Knowledge, Attitudes, and Practices About Parasitic Diseases and AR

We asked the respondents about signs associated with parasitic diseases in eight separate questions. The most common responses were emaciation (146), general weakness (121), low appetite (117), diarrhea (111), fever (56), abnormal wool/hair coat (42), icterus (26), and abortion/still birth (15). Fourty-three percent of the Hamedan farmers knew that parasites developed resistance because of non-principled use of anthelmintics; 43.2% considered that rotation of anthelmintics could prevent emergence of AR; and 83.9% were aware that shared pasture grazing can lead to transmission of parasites among different flocks. Less than 50% of the farmers were aware of zoonotic transmission of parasites from livestock (Table 3).

**Table 3:** Knowledge, attitudes, and practices of farmers (*n* = 150) in the study regarding parasitic diseases and AR in Hamedan province, 2019.

**Knowledge**	**True**	**False**	**Do not know**
• Helminths develop resistance because of non-principled use of anthelmintics	43.0	2.7	54.0
• One drug can be used for several types of helminth infections	54.1	33.1	12.8
• Newley arrived livestock should be quarantined for a reasonable period of time.	75.8	2.0	22.1
• Up to 67% of flocks might have livestock with worms resistant to levamisole and albendazole	12.2	2.0	85.8
• Rotational use of anthelmintics could prevent emergence of AR	43.2	0	56.8
• Sheep, goats and cattle might share some parasites	88.6	0	11.4
• Milk and meat of livestock should not be used for a period after anthelmintic treatment	52.3	4.0	43.6
• Some helminth infections are zoonotic	43.9	1.4	54.7
• Shared pasture grazing can lead to transmission of parasites among different flocks	83.9	8.7	7.4
**Attitudes**	**Agree**	**No idea**	**Disagree**
• AR is a serious problem in Iran	36.5	60.1	3.4
• One of the main causes of AR is application of non-prescribed anthelmintics	60.7	36.0	3.3
• Manufacturers' instructions should be read for every drug	96.7	2.6	0.7
• It is good to consult with a veterinarian before deciding any treatment	99.3	0	0.7
• More expensive anthelmintics work better^a^	51.0	9.4	39.6
• Imported anthelmintics work better in comparison with domestic products^a^	59.3	12.0	28.7
**Practices**	**Always/usually**	**Some times**	**Rarely/never**
• I consult my veterinarian regarding anthelmintic treatment	48.7/44.7	3.3	0/0
• I treat the whole flock upon observation of general signs of helminth diseases^b^	39.3/51.7	8.3	0.7/0
• I send fecal samples to the laboratory for diagnosis of helminths	0.7/1.4	1.4	4.1/92.4
• I read manufacturers' instructions for every drug before its application	13.9/16.7	8.3	30.6/30.6
• I use all types of anthelmintics (liquid, bolus, injectable)^b^	38.7/46.0	10.0	2.7/0
• I consider meat and milk withdrawal time after anthelmintic treatment	8.2/13.7	16.4	40.4/21.2
• I will treat my flock if helminth parasitism is diagnosed by veterinarian in a neighboring flock^b^	39.3/44.8	6.79	6.2/2.8
• I use same drug for treatment of different diseases with similar signs^b^	5.5/56.8	29.5	6.2/2.1
• I quarantine newly bought livestock	6.8/7.5	14.4	25.3/45.9
• I feed dogs and cats with infected offal with cysts and parasites^b^	3.3/8.0	24.0	22.0/38.7

Numbers are in percentage.

There were a few cases of missing values for some questions. Only valid percentages are presented in the table.

a Except these two statements, other items in the Attitudes section were regarded as positive attitudes.

b *Scoring in the Practices section was 4, 3, 2, 1, and 0 for always, usually, sometimes, rarely and never for all items, except these five statements which reverse scoring was used for them*.

**Figure 1:**
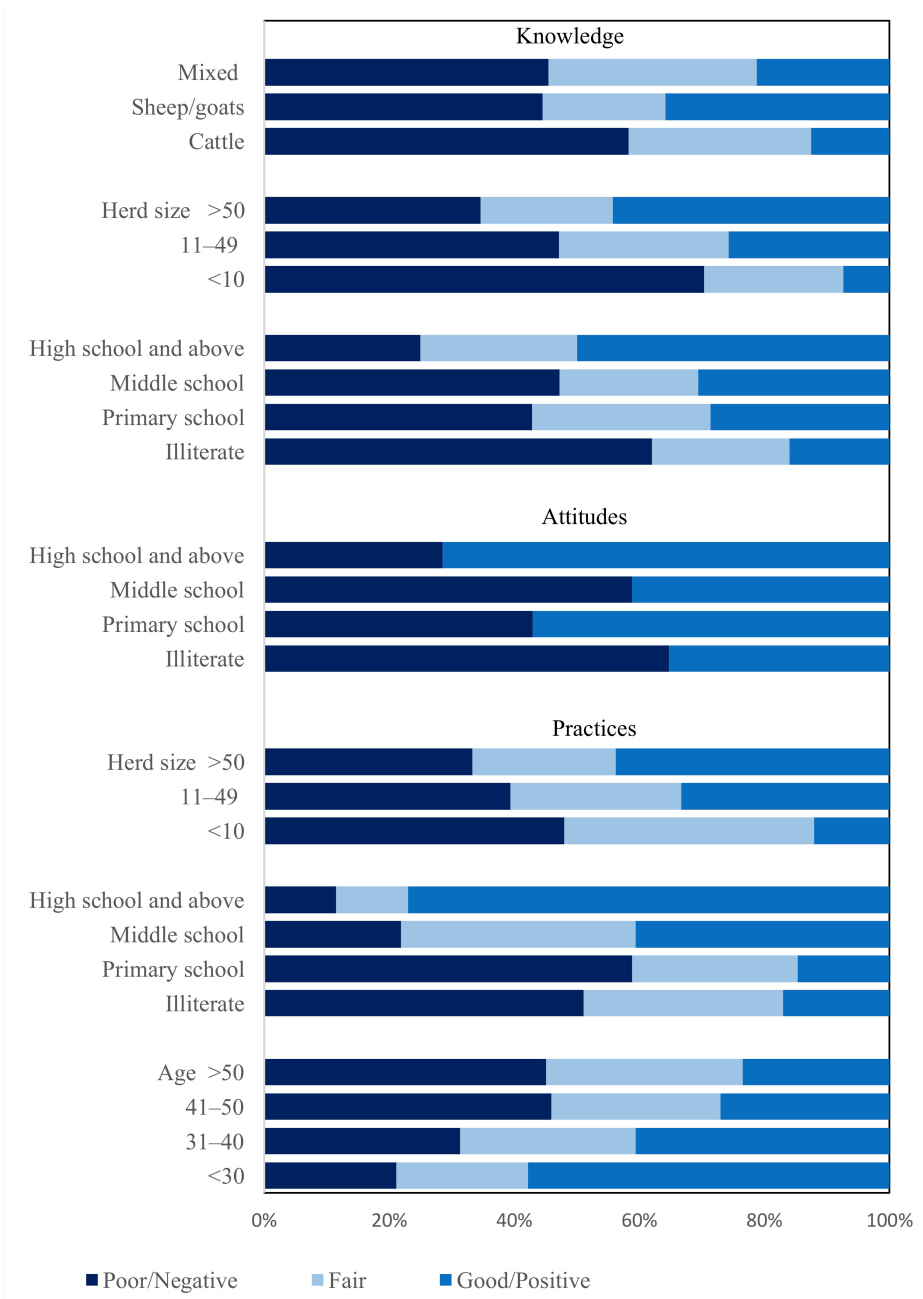
Distribution of knowledge, attitudes and practices according to various explanatory variables for farmers (*n* = 150) in the study regarding parasitic diseases and anthelmintic resistance in Hamedan province, 2019.

The range (minimum to maximum) for total knowledge scores was 2 to 17 (mean ± SD: 8.2 ± 3.5). According to the total score, 28.39% of the respondents had good knowledge of parasitic diseases (total score 11 to 17) and corresponding measures for the fair (total score 8 to 10) and poor (total score <8) knowledge groups were 24.2 and 47%, respectively. In univariable ordinal logistic regression analyses for knowledge groups, education (*P* = 0.042), herd size (*P* = 0.007) and livestock type (*P* = 0.114) had *P* < 0.2, and were introduced into the multivariable model (Figure 1). The results showed that education and herd size had significant associations with knowledge groups and remained in the final model. Livestock type (*P* = 0.857) was not retained in the final model. Based on the odds ratios in the final model, less educated farmers and farmers with smaller herd size were more likely to be in the poor knowledge groups compared to more educated farmers and farmers with larger herd size (Table 4).

**Table 4:** Results of multivariable ordinal logistic regression analyses for factors associated with knowledge, attitudes and practices of farmers (*n* = 150) in the study regarding parasitic diseases and AR in Hamedan province, 2019.

**Parameters**	**β**	**SE**	**Odds ratio**	**95% Confidence Interval**	***P*-value**
**Knowledge**^**a**^					
Constant 1	−1.588	0.428	–	–	–
Constant 2	−0.428	0.406	–	–	–
**Education**					
Illiterate	−1.466	0.474	0.231	0.092, 0.578	0.002
Primary school	−0.796	0.490	0.451	0.174, 1.167	0.101
Middle school	−0.799	0.485	0.450	0.172, 1.176	0.103
High school and above (ref)	–	–	–	–	–
**Herd size**					
<10	−1.568	0.541	0.208	0.078, 0.556	0.002
11–49	−0.590	0.352	0.554	0.276, 1.114	0.098
>50 (ref)	–	–	–	–	–
**Attitudes**^**b**^					
Constant 1	0.588	0.394	–	–	–
**Education**					
Illiterate	−1.194	0.491	0.303	0.116, 0.794	<0.001
Primary school	−0.300	0.522	0.741	0.266, 2.060	0.053
Middle school	−0.944	0.526	0.389	0.139, 1.091	0.013
High school and above (ref)	–	–	–	–	–
**Practices**^**c**^					
Constant 1	−2.592	0.494	–	–	–
Constant 2	−1.138	0.454	–	–	–
**Education**					
Illiterate	−2.657	0.550	0.070	0.023, 0.210	<0.001
Primary school	−2.939	0.586	0.053	0.016, 0.170	<0.001
Middle school	−1.447	0.563	0.235	0.077, 0.719	0.011
High school and above (ref)	–	–	–	–	–

a Model information for knowledge in three groups, poor, fair, and good: −2log Likelihood change = 23.60, df = 5, P < 0.001; Goodness of fit Pearson chi-square = 28.04, df = 17, P = 0.044, Test of parallel lines chi-square = 3.801, df = 5, P = 0.578.

b Model information for attitudes in two groups, positive and negative attitudes: −2log Likelihood change = 8.14, df = 3, P = 0.043;

c *Model information for practices in three groups, poor, fair, and good: −2log Likelihood change = 37.626, df = 3, P < 0.001; Goodness of fit Pearson chi-square = 1.601, df = 3, P = 0.659; Test of parallel lines chi-square = 1.534, df = 3, P = 0.674*.

The results of the attitudes section showed that among 150 farmers, 36.5% believed that AR is a serious problem, and 60.7% agreed that self-administration of anthelmintics plays a key role in its emergence. Interestingly, more than half of the farmers believed that “more expensive” and “foreign-branded” drugs work better (Table 3). The minimum of total attitudes score was −3 and the maximum was 6 (mean ± SD: 2.42 ± 2). According to the total score, 47.3% of the respondents were in the positive attitudes group (total score ≥3), and 52.7% were in the negative attitudes groups (total score <3). Only education (*P* = 0.045) was introduced to the binary logistic regression model for attitudes (Figure 1). The results showed that farmers with higher levels of education were more likely to be in the positive attitudes group (Table 4).

With regard to practices, almost all of the farmers reported that they frequently consulted with a veterinarian about anthelmintic treatments. Whole flock treatments were frequently practiced, and fecal samples were rarely collected to estimate helminth burdens. Only 30.6% of the farmers read the drug manufacturers' instructions, and only 21.9% adhered to meat and milk withdrawal times after anthelmintic treatment. Throwing infected offal with cysts and parasites for dogs and cats was practiced to some extent by 61.3% of farmers (Table 3). According to the total scores, 44% of the respondents adopted poor practices in regards to preventing parasitic infections and AR mitigation. Total practices scores ranged from 7 to 31 (mean ± SD: 15.3 ± 4.4). A total of 33.1% of farmers were in the good practices group (total score 17 to 31), 28.1% were in the fair group (total score 14–16) and 38.8% were in the poor group (total score <14). Age (*P* = 0.149), education (*P* < 0.001), and herd size (*P* = 0.105) were associated with practices groups in the univariable analysis (Figure 1). Using multivariable ordinal logistic regression analysis, only education showed a significant relationship with practices in the final model (Table 4). Farmers with higher education were more likely to be in the better practices groups.

Spearman's rank correlation coefficients showed that total knowledge score have significant positive associations with both attitudes (r_s_ = 0.20, *P* = 0.015) and practices scores (r_s_ = 0.29, *P* < 0.001). The correlation between attitudes and practices scores was not significant (r_s_ = 0.15, *P* = 0.079). The calculated Cronbach's alpha for items in the knowledge, attitudes, and practices modules were 0.67, 0.48, and 0.61, respectively.

The results of the additional questions on practices showed that feeding livestock with medicinal plants for anthelmintic treatment was uncommon; and that only 50% of the 92 farmers who kept sheepdogs, treated them for helminths. Albendazole (99%) and ivermectin (83%) were the most commonly used anthelmintic drugs, followed by niclosamide (68.5%), rafoxanide (68.3%), levamisole (61.8%), closantel (55.2%), and praziquantel (33.8%). Most farmers declared they practiced anthelmintic treatment in their flocks or herds only when recommended by a veterinarian (71%), while others reported using these drugs with various regular intervals in each year, for example once a year (19.4%), twice a year (14.6%), or four times a year (5.4%). Also, 15.4% of the farmers reported that they treated their livestock whenever they considered it to be necessary. In response to the question *what will you do if you realize that the administered anthelmintic is not effective*, 95% of the respondents answered that they would consult with a veterinarian. Responses also included increasing the dose (36.7%), changing the drug (22.7%), and slaughtering or selling the animals (11.4%). In this section totals could exceed the number of enrolled farmers because options were not exclusive choices.

## Discussion

Obtaining insights into factors that drive farmers' decisions is necessary in order to develop sustainable parasite control strategies. In this study, KAP of farmers in Hamedan province, Iran regarding parasitic diseases and AR mitigation was assessed. Chronbach's alpha was calculated for each section concerning knowledge, attitudes and practices to ensure consistency. According to a wide range of different qualitative descriptors which was used to interpret the Chronbach's alpha values (15), the values were reasonable for knowledge, satisfactory for practices and acceptable for attitudes.

Farmers in this survey were mostly middle-aged males with relatively low education, consistent with the general demographics of livestock keepers in Asia and Africa (16, 17). Education level had a significant association with farmers' awareness about AR, as well as their knowledge, attitudes and practices toward sustainable worm control. Farmers who kept the most livestock were better informed about the concept of AR mitigation. However, age of farmers, years of husbandry experience and type of livestock were not associated with their understanding of AR. In contrast, in a recent study from Iran that assessed KAP of small ruminant farmers, an association was observed between experience and the total number of animals each farmer owned, and understanding regarding several infectious diseases (17). This may imply that Iranian farmers are more familiar with visible clinical signs in their livestock (for example, mortality, lameness, skin lesions, etc.) than with subclinical losses (for example, resulting from helminth parasitism as a result of poor drug efficacy). More than half of the farmers in the present study had heard about AR from other farmers, but most expressed an interest in working with veterinarians to share reliable information and advice regarding anthelmintic treatments. Low levels of education in farmers is considered to be a serious constraint in effective worm control (5). Agriculture-related education can positively influence perceptions of AR risk (7), and in the present study, extension courses of the Ministry of Agriculture were a principal source of information for farmers. It is, therefore, recommended that regular country-wide classes should be held to educate farmers on the evidence-based principles of sustainable helminth control.

Herd size had significant positive associations with the total knowledge scores of farmers of Hamedan and with their awareness regarding parasites and AR. Consistent with our results, in a recent study from Iran, owners of larger flocks and herds had considerably better understanding of enterotoxaemia, sheep and goat pox, and foot-and-mouth disease (17). Larger herd and flock sizes are usually associated with higher stocking densities, increased pasture helminth larval contamination, a higher parasite infection pressure, and more frequent anthelmintic treatments. In a Canadian study, sheep flock size was not considered to be a risk factor for AR (18), but most of the flocks would have been larger than those in the current study, and their grazing management would have differed with regards to helminth infection pressures.

Less than half of the farmers were aware of zoonotic transmission of parasites from livestock although infection of humans with *Trichostrongylus* and *Fasciola* species is regularly reported from different regions of Iran (19, 20). Throwing infected offal with cysts and parasites for dogs and cats was practiced to some extent by 61.3% of farmers, and only 50% of the farmers in this study treated their sheepdogs for helminths. Canine-borne metacestodes of *Taenia* species i.e., cysticercus ovis, cysticercus tenuicollis, and coenurus cerebralis, as well as sarcocystosis, are prevalent in livestock of different regions of the country (21, 22) with no estimation of their direct (condemnation of infected organs and carcasses) and indirect (production losses) economic complications. However, the most important zoonotic disease in Iran with an estimated annual monetary burden of US$232.3 million is cystic echinococcosis (23). The weighted prevalence of hydatidosis in animal and human intermediate hosts reach 15.6 and 5%, respectively (24, 25). Livestock in Iran are mainly slaughtered in industrial abattoirs with standardized protocols for condemnation of infected organs, but farmers in remote areas slaughter their own sheep and goats. Our findings highlight the need for public health education and knowledge dissemination on zoonotic diseases.

More than half of the farmers in Hamedan had a perception that more expensive and “foreign-branded” anthelmintics work better. Interestingly, it has been shown that consumers often believe and, therefore, judge lower-priced items to be of lower quality (26). Nevertheless, studies of the comparative efficacy of local and “foreign branded” albendazole (10) and levamisole (9) in Iran revealed no significant difference between them. However, despite high levels of nationalism and preference for indigenous manufacturers, attitudes of Iranian consumers toward buying “foreign-made” products have been reported (27, 28). In recent years with increases in economic sanctions imposed on Iran, products of internationally well-known pharmaceutical companies have greatly decreased in the market. Based on the author's experience, livestock farmers are willing to pay more for “foreign-branded” drugs that are sometimes low quality or are fake. It is necessary to regularly test the efficacy of Iranian branded drugs in the field to bring trust to farmers.

In the present study over 60% of farmers stated that they neither read anthelmintic drug manufacturers' instructions before administration, nor considered meat and milk withdrawal periods. This is a common problem in developing countries around the world, for example in Tanzania where cattle farmers did not observe withdrawal periods in anthelmintic treated animals (5). Each anthelmintic of ruminants has a certain withdrawal period time within which the drug and its metabolites are deposited in meat and secretions of the livestock such as milk and should not be consumed (29). In Iran determination of antibiotic residues in milk is not obligatory by government, but as majority of dairy companies request daily results from laboratories, farmers are careful not to sell milk after administration of antibiotics. However, although hazardous effects of anthelmintics for human health has been shown (30), there are no regulations in Iran for monitoring residues of commonly used drugs such as albendazole and ivermectin in milk and meat.

Quarantine was never or rarely practiced by 71.2% of farmers in this study, although one of the recommendations for sustainable helminth control including AR mitigation is implementing an effective quarantine strategy (31). It is recommended that new animals should be drenched with an effective anthelmintic and released to the common pastures after a fecal egg count reduction test (3). However, the costs of this may not be justified within the economic framework of livestock farming, highlighting the need to present sustainable helminth control practices in a way that can be practically integrated into farmers' businesses.

Finally, as a limitation of the present study, it should be noted that bias in questionnaire surveys is an important issue in public health research and has three main sources: design of each question; design of a questionnaire as a whole; and the way the questionnaire is administered (32). In the present study, we tried to minimize bias by carefully designing each question and pre-testing the questionnaire using both farmers and veterinarians as respondents. However, in the knowledge section of the questionnaire, all “true” options were correct answers and this may be a potential source of response bias. Furthermore, it should be acknowledged that some sources of bias such as response bias due to self-reported data may be inevitable or difficult to control. It has been shown that this type of bias may occur in self-reported attitudes and practices for various reasons such as when the participant wants to look good in the survey, even if the survey is anonymous (33). The results of the present work, therefore, may suffer from social desirability bias.

In summary, the results of this study show that although the knowledge and attitudes of most of the farmer participants in Hamedan province, Iran were acceptable; 44% adopted poor practices regarding parasite control and AR mitigation. This discrepancy between knowledge and practice raises various challenges and opportunities, for example surrounding the use of non-prescribed anthelmintics, monitoring of parasitic infections, and considering drug withdrawal periods. The study highlights the principle that increased knowledge and positive attitudes do not necessarily result in positive change in farmers' behaviors. Sustainable helminth control strategies in Iran must take into account the factors that govern why farmers' make decisions concerning their businesses. More farm veterinarians with relevant interests and expertise are needed as influential sources of advice on sustainable helminth control. Regulatory supervision of anthelmintic drug sales to farmers would be helpful in this process of knowledge and understanding transfer.

## Data Availability Statement

All datasets generated for this study are included in the article/Supplementary Material.

## Ethics Statement

Participants were provided with an information describing the team, study objectives, and they were reassured that all responses will be anonymous.

## Author Contributions

AS: conceptualization, writing—original draft preparation, supervision, project administration, and funding acquisition. AS, SZa, GA, and SZo: methodology. NS and AA: writing—review and editing. All authors read and approved the final manuscript.

## Conflict of Interest

The authors declare that the research was conducted in the absence of any commercial or financial relationships that could be construed as a potential conflict of interest.
