# Supplementary material for: Assessment of Knowledge, Attitudes and Practices Relating to Parasitic Diseases and Anthelmintic Resistance Among Livestock Farmers in Hamedan, Iran
Source: Front Vet Sci. 2020 Oct 26;7:584323. doi: 10.3389/fvets.2020.584323 (PMC7649137; doi:10.3389/fvets.2020.584323)
Supplement: Supplementary file 1 [file Data_Sheet_1.doc]

**Supplementary material**

**Questionnaire for livestock farmers**

| Date__________ | Questionnaire number __________ |
| --- | --- |
| Region of husbandry __________ | Investigator___________ |

Hello! We are investigators from Bu-Ali Sina University. We aim to understand your knowledge, attitude and practice regarding parasitic diseases and anthelmintic resistance. **The survey is anonymous and your name is not required.** Your information collected by the survey is only for the use of scientific research and will not be disclosed to irrelevant people. Your answers are very important for us but this survey is volunteered. This interview will take 5-10 minutes. We are very appreciated for your participation and support!

For your information, resistance means is the ability of the worm to survive treatment with an anthelmintic.

**Part I Basic information**

1. Gender [A] male [B] female

2. Date of birth _______________________________

3. Education background:

| [A] illiterate | [B] elementary school | [C] intermediate school | [D] high school or above |
| --- | --- | --- | --- |

4. How many years are you engaged in livestock farming-related work? _____________________years.

5. How many and what type of livestock do you have?

 cattle number__________

 sheep/goat number__________

 camel number__________

 horse number__________

6. Type of husbandry [A] grazing [B] stall feeding [C] grazing plus stall feeding

7. Have you ever heard about anthelmintic resistance? [A] yes [B] no

8. If yes, how did you gained this knowledge?

| [A] Ministry of Agriculture extension courses | [B] veterinarian | [C] TV/radio |
| --- | --- | --- |
| [D] book/journal/leaflets/booklet | [E] internet/social media | [F] other farmers |

9. Are you willing to learn more about the concept of anthelmintic resistance? [A] yes [B] no

10. If yes, from which source do you like to get the information?

| [A] Ministry of Agriculture extension courses | [B] veterinarian |
| --- | --- |
| [C] TV or radio | [D] book/journal/leaflets/booklet |
| [E] internet and social media | [F] other: please specify__________ |

**Part II Knowledge about parasitic diseases and anthelmintic resistance**

Which of these signs are associated with parasitic diseases?

| 1. emaciation | [A] yes | [B] no | [C] I don’t know |
| --- | --- | --- | --- |
| 2. diarrhoea | [A] yes | [B] no | [C] I don’t know |
| 3. icterus | [A] yes | [B] no | [C] I don’t know |
| 4. fever | [A] yes | [B] no | [C] I don’t know |
| 5. abortion and still birth | [A] yes | [B] no | [C] I don’t know |
| 6. abnormal wool/hair coat | [A] yes | [B] no | [C] I don’t know |
| 7. low appetite | [A] yes | [B] no | [C] I don’t know |
| 8. general weakness | [A] yes | [B] no | [C] I don’t know |

**Define the true or false.**

| 9. Helminths develop resistance because of non-principled use of anthelmintics | [A] true | [B] false | [C] I don’t know |
| --- | --- | --- | --- |
| 10. One drug can be used for several types of helminth infections | [A] true | [B] false | [C] I don’t know |
| 11. Newley arrived livestock should be quarantined for a reasonable period of time | [A] true | [B] false | [C] I don’t know |
| 12. Up to 67% of flocks might have livestock with worms resistant to levamisole and albendazole | [A] true | [B] false | [C] I don’t know |
| 13. Rotational use of anthelmintics could prevent emergence of AR | [A] true | [B] false | [C] I don’t know |
| 14. Sheep, goats and cattle might share some parasites | [A] true | [B] false | [C] I don’t know |
| 15. Milk and meat of livestock should not be used for a period after anthelmintic treatment | [A] true | [B] false | [C] I don’t know |
| 16. Some helminth infections are zoonotic | [A] true | [B] false | [C] I don’t know |
| 17. Shared pasture grazing can lead to transmission of parasites among different flocks | [A] true | [B] false | [C] I don’t know |

**Part III Attitude**

| 1. AR is a serious problem in Iran | [A] agree | [B] disagree | [C] no idea |
| --- | --- | --- | --- |
| 2. One of the main causes of AR is application of non-prescribed anthelmintics | [A] agree | [B] disagree | [C] no idea |
| 3. Manufacturers’ instructions should be read for every drug | [A] agree | [B] disagree | [C] no idea |
| 4. It is good to consult with a veterinarian before deciding any treatment | [A] agree | [B] disagree | [C] no idea |
| 5. More expensive anthelmintics work better | [A] agree | [B] disagree | [C] no idea |
| 6. Imported anthelmintics work better in comparison with domestic products | [A] agree | [B] disagree | [C] no idea |

**Part IV Practice**

1. I consult my veterinarian regarding anthelmintic treatment

[A] always [B] usually [C] some times [D] rarely [E] never

2. I treat the whole flock upon observation of general signs of helminth diseases.

[A] always [B] usually [C] some times [D] rarely [E] never

3. I send faecal samples to the laboratory for diagnosis of helminths

[A] always [B] usually [C] some times [D] rarely [E] never

4. I read manufacturers’ instructions for every drug before its application

[A] always [B] usually [C] some times [D] rarely [E] never

5. I use all types of anthelmintics (liquid, bolus, injectable)

[A] always [B] usually [C] some times [D] rarely [E] never

6. I consider meat and milk withdrawal time after anthelmintic treatment

[A] always [B] usually [C] some times [D] rarely [E] never

7. I will treat my flock if helminth parasitism is diagnosed by veterinarian in a neighbouring flock

[A] always [B] usually [C] some times [D] rarely [E] never

8. I use same drug for treatment of different diseases with similar signs

[A] always [B] usually [C] some times [D] rarely [E] never

9. I quarantine newly bought livestock

[A] always [B] usually [C] some times [D] rarely [E] never

10. I feed dogs and cats with infected offal with cysts and parasites

[A] always [B] usually [C] some times [D] rarely [E] never

11. Do you treat your dog with anthelmintics? [A] I don’t have a dog [B] yes [C] no

12. Which of the following anthelmintic drugs have you used?

|  | Generic name | Brand name | Available forms |
| --- | --- | --- | --- |
| 1 | Albendazol | Diverm, Albazan | bolus/tablet, suspension |
| 2 | Rafoxanide | Rafoxan | bolus/tablet, suspension |
| 3 | Ivermectin | Ivectin, Erfamectin | injectable, oral solution |
| 4 | Niclosamide | Nicolsam | bolus/tablet |
| 5 | Praziquantel | Droncit, Lorencit | bolus/tablet, suspension |
| 6 | Levamisole | Loramisole, Vetamisole | powder, suspension, injectable |
| 7 | Closantel | Hepatec, Closa, Closal | bolus/tablet |
| 8 | Buparvaquone | Butalex, Vetalex | injectable |

13. how often they used anthelmintic in their flocks or herds?

[A] every 3 months [B] every 6 months [C] every year [D] when the veterinarian will advise

[E] when I consider it is necessary

14. what will you do if you realise that the administered anthelmintic is not effective?

[A] I will increase the dose [B] I will consult with a veterinarian [C] I will change the drug

[D] I will slaughter or sell the animal

15. Do you use medicinal plants for treatment of parasitic diseases? [A] no [B] yes, it is__________
